# A role for antizyme inhibitor in cell proliferation

**DOI:** 10.1007/s00726-015-1957-6

**Published:** 2015-03-27

**Authors:** Tania M. Silva, Helena Cirenajwis, Heather M. Wallace, Stina Oredsson, Lo Persson

**Affiliations:** 1 Department of Biology, Lund University, Lund, Sweden; 2 Division of Applied Medicine, University of Aberdeen, Polwarth Building, Foresterhill, Aberdeen, UK; 3 Department of Experimental Medical Science, Lund University, Lund, Sweden; 4 Present Address: Laboratory of Microbiology and Immunology of Infection, Institute for Molecular and Cell Biology, Porto University, Porto, Portugal; 5 Present Address: Division of Oncology and Pathology, Department of Clinical Sciences, Lund University, Lund, Sweden

**Keywords:** Antizyme inhibitor 1, Polyamines, Ornithine decarboxylase, Cell proliferation, Breast cancer

## Abstract

The polyamines are important for a variety of cellular functions, including cell growth. Their intracellular concentrations are controlled by a complex network of regulatory mechanisms, in which antizyme (Az) has a key role. Az reduces the cellular polyamine content by down-regulating both the enzyme catalysing polyamine biosynthesis, ornithine decarboxylase (ODC), and the uptake of polyamines. The activity of Az is repressed by the binding of a protein, named Az inhibitor (AzI), which is an enzymatically inactive homologue of ODC. Two forms of AzI have been described: AzI1, which is ubiquitous, and AzI2 which is expressed in brain and testis. In the present study, we have investigated the role of AzI1 in polyamine homeostasis and cell proliferation in breast cancer cells. The results obtained showed that the cellular content of AzI increased transiently after induction of cell proliferation by diluting cells in fresh medium. Inhibition of polyamine biosynthesis induced an even larger increase in the cellular AzI content, which remained significantly elevated during the 7-day experimental period. However, this increase was not a consequence of changes in cell cycle progression, as demonstrated by flow cytometry. Instead, the increase appeared to correlate with the cellular depletion of polyamines. Moreover, induced overexpression of AzI resulted in an increased cell proliferation with a concomitant increase in ODC activity and putrescine content. During mitosis, AzI1 was localised in a pattern that resembled that of the two centrosomes, confirming earlier observations. Taken together, the results indicate that AzI fulfils an essential regulatory function in polyamine homeostasis and cell proliferation.

## Introduction

Polyamines are recognised as necessary growth factors in all cells, including cancer cells (Wallace et al. 2003; Gerner and Meyskens 2004; Nowotarski et al. 2013). Perturbation of intracellular polyamine content in mammalian cells has dramatic effects on cell proliferation and cell death/apoptosis. Thus, a complex of elaborate mechanisms, affecting synthesis, degradation as well as membrane transport, has evolved for the control of polyamine homeostasis (Persson 2009; Pegg and Casero 2011). Ornithine decarboxylase (ODC) is a key regulatory protein catalysing the first and rate-limiting step in polyamine biosynthesis. This enzyme is strictly regulated by antizyme (Az), which binds to ODC with a high affinity at the monomeric level, inhibiting the formation of the active ODC homodimer and targeting it for ubiquitin-independent degradation by the 26S proteasome (Kahana 2009; Murai et al. 2011).

In addition, Az inhibits the cellular uptake of extracellular polyamines (Mitchell et al. 1994; Suzuki et al. 1994). The cellular content of Az is partly regulated by polyamines, which stimulate its synthesis by a unique mechanism involving ribosomal frame-shifting (Rom and Kahana 1994; Matsufuji et al. 1995). At least three different mammalian isoforms of Az (Az1–Az3) have been identified, of which Az1 and Az2 are ubiquitously expressed, whereas Az3 is testis-specific (Kahana 2009). All three Az isoforms are capable of inhibiting ODC activity and polyamine uptake, but only Az1 and Az2 can target ODC for degradation (Kahana 2009).

In addition to Az, cells contain another protein with a putative role in polyamine homeostasis. This protein was first discovered in rat liver by the potential to reactivate Az-inhibited ODC and was thus given the name of Az inhibitor (AzI) (Fujita et al. 1982). AzI, which is an enzymatically inactive homologue of ODC, binds to Az with higher affinity than does ODC and consequently releases ODC from its inactive ODC-Az complex (Fujita et al. 1982). By binding to Az, AzI also prevents the effects of Az on ODC degradation and cellular uptake of polyamines (Kahana 2009; Murai et al. 2011). Keren-Paz et al. (2006) demonstrated that NIH3T3 cells overexpressing AzI had increased ODC activity and polyamine uptake, and exhibited improved cell proliferation. Moreover, these cells gave rise to tumours when injected into nude mice (Keren-Paz et al. 2006), whereas knockdown of AzI using siRNA or shRNA decreased cell proliferation both in vitro and in vivo (Choi et al. 2005; Keren-Paz et al. 2006; Olsen et al. 2012). Mutant mice with both AzI alleles disrupted, died close to birth and displayed abnormal liver morphology and perturbed polyamine homeostasis (Tang et al. 2009). Interestingly, AzI has also been shown to be up-regulated in a number of human cancers (Jung et al. 2000; Schaner et al. 2003; van Duin et al. 2005; Chin et al. 2007; Olsen and Zetter 2011).

In spite of its putative role in polyamine homeostasis and its cell proliferation-promoting effects, information on the regulation and cellular function of AzI is relatively sparse. Nevertheless, a close correlation between cell proliferation, ODC and AzI has been demonstrated (Murakami et al. 1989; Nilsson et al. 2000; Murakami et al. 2009). Results indicate that AzI may have other functions besides being involved in polyamine homeostasis. Zetter and colleagues reported that AzI reduced the turnover of cyclin D, as well as induced centrosome overduplication (Kim et al. 2006; Mangold et al. 2008). A co-localisation of Az1 and AzI to the centrosome during mitosis was confirmed by Murakami et al. (2009). In addition to the ubiquitously present AzI, a closely related protein, termed AzI2, is expressed in brain and testis (Pitkanen et al. 2001; Lopez-Contreras et al. 2006). Thus, the first AzI discovered is sometimes referred to as AzI1. Similar to AzI1, AzI2 neutralises the effects of Az on ODC and the cellular uptake of polyamines (Lopez-Contreras et al. 2008; Snapir et al. 2008). In addition, Kanerva et al. (2010) presented results indicating that AzI2 is involved in the regulation of vesicular transport within the cell.

In the present study, we investigated the role of AzI1 (referred to as AzI) in polyamine homeostasis and cell proliferation in breast cancer cells. The results show that AzI expression varied in relation to cell proliferation and polyamine content. Moreover, induced overexpression of AzI resulted in an increased cell proliferation with a concomitant increase in ODC activity and putrescine content. AzI was shown to be localised in a centrosomal pattern during mitosis, confirming earlier observations of a co-localisation of AzI with centrosomes (Mangold et al. 2008; Murakami et al. 2009).

## Materials and methods

Cell culture medium components were purchased from Biochrom, Berlin, Germany. Tissue culture plastics were acquired from Nunc, Roskilde, Denmark. Phosphate-buffered saline (PBS: 8 g/L NaCl, 0.2 g/L KCl, 1.15 g/L Na_2_HPO_4_, 0.2 g/L KH_2_PO_4_, pH 7.3) was purchased from Oxoid Ltd., Basingstoke, Hampshire, UK. Nonidet P-40 was purchased from VWR, Lund, Sweden. Insulin and propidium iodide (PI) were obtained from Sigma, Stockholm, Sweden. Dimethyl sulphoxide (DMSO) was acquired from Merck KGaA, Darmstadt, Germany. l-[1-^14^C] Ornithine (1.9 GBq/mmol) was purchased from New England Nuclear Du Pont, Scandinavia AB, Stockholm, Sweden. Bromodeoxyuridine (BrdUrd) and the primary monoclonal antibody against BrdUrd (Clone: Bu20a), as well as the secondary fluorescein isothiocyanate (FITC)-conjugated antibody and the secondary horseradish peroxidase-conjugated goat anti-mouse IgG antibody, were all purchased from DAKO, Glostrup, Denmark. Hybond enhanced chemiluminescence (ECL) nitrocellulose membrane and ECL™ Advance Blotting Detection Kit (Amersham Biosciences) were purchased from GE Healthcare, Uppsala, Sweden. Geneticin and all components of the NuPAGE Novex Pre-Cast Gel System used for Western blot were obtained from Invitrogen Corporation, Carlsbad, CA, USA. The antibody against β-actin was purchased from Abcam, Cambridge, UK. The monoclonal antibody against rat AzI1 was kindly provided by Dr. Senya Matsufuji and Dr. Yasuku Murakami, Tokyo, Japan. Gene Pulser Cyvettes (0.4 and 0.1 cm) were purchased from Bio-Rad, Hercules, CA, USA. The mammalian expression vector pCl-neo was purchased from Promega Corporation, Madison, WI, USA. The Nucleo Spin Extract Kit was obtained from Clonetech Laboratories, Inc., Mountainview, CA, USA. The agarose gels were obtained from Bio-Rad Laboratories, Hercules, CA, USA. The Genelute HP plasmid midi-prep kit was purchased from Sigma-Aldrich Sweden AB, Stockholm, Sweden. Restriction enzymes and T4 DNA ligase were purchased from Fermentas GMBH, Helsingborg, Sweden. The oligos for PCR were obtained from TAG, Copenhagen, Denmark. Difluoromethylornithine (DFMO) was purchased from Ilex-Oncology, San Antonio, Texas, USA. SAM486A was a kind gift from Novartis, Basel, Switzerland.

### Drug stock solutions

Stock solutions of SAM486A (2 mM), aminoguanidine (AG, 50 mM), putrescine (50 mM) and spermidine (50 mM) were made in PBS. DFMO was dissolved in Millipore water to obtain a concentration of 0.5 M after adjusting pH to 7.2. All solutions were sterile-filtered and used in the experiments at final concentrations of: SAM486A (20 μM), AG (1 mM), putrescine (100 μM), spermidine (50 μM) and DFMO (1 mM).

### Cell culture

The human breast carcinoma cell line JIMT-1 (ACC589) was purchased from the German Collection of Microorganisms and Cell Cultures, DSMZ (Braunschweig, Germany) and was cultured in DMEM/Ham’s F12 medium supplemented with 10 % foetal bovine serum, non-essential amino acids (1 mM), insulin (10 µg/ml), penicillin (100 U/ml) and streptomycin (100 µg/ml). The human breast carcinoma cell line MCF-7 (HTB22) was obtained from the American Type Culture Collection (Manassas, VA, USA) and was cultured in RPMI 1640 medium supplemented with 10 % foetal bovine serum, non-essential amino acids (1 mM), insulin (10 µg/ml), penicillin (100 U/ml) and streptomycin (100 µg/ml). Both cell lines were cultured as monolayers at 37 °C in a humidified incubator with 5 % CO_2_ in air. The JIMT-1 cell line, having a doubling time of approximately 24 h, was sub-cultured twice a week, while the MCF-7 cell line, with a doubling time of approximately 34 h, was sub-cultured once a week. In all experiments, plateau phase cells were reseeded to a lower cell density (30,000 cells/cm^2^). The cells were seeded in the absence or presence of DFMO, SAM486A, DFMO/putrescine or DFMO/spermidine. In experiments using spermidine, AG was added to inhibit any activity of polyamine oxidases present in the bovine serum. The cells were harvested by trypsinisation and the cell number was determined by counting in a hemocytometer at times indicated in the figures. The cells were pelleted and stored at −80 °C until further analysis.

### ODC activity assay

The cells were sonicated in ice-cold 0.1 M Tris–HCl (pH 7.5) containing 0.1 mM EDTA and 2.5 mM dithiothreitol. The ODC activity was determined by measuring the release of [^14^C] CO_2_ from carboxyl-labelled l-ornithine, in the presence of saturating levels of pyridoxal 5-phosphate (0.1 mM) and l-ornithine (0.2 mM) (Jänne and Williams-Ashman 1971).

### Polyamine analysis

Chromatographic separation and quantitative determination of the polyamines in cell extracts in 0.2 M perchloric acid were carried out using a HPLC (Hewlett Packard 1100) with *θ*-phthaldialdehyde as the reagent essentially as previously described (Seiler and Knödgen 1980).

### Cell cycle progression analysis

Cells were seeded in the absence or presence of DFMO and harvested by trypsinisation every second hour for up to 24 h after seeding. Thirty minutes before harvesting, BrdUrd (5 µM) was added to the medium of the cells. The cells were collected and fixed in ice-cold 70 % ethanol and stored at −20 °C until further analysis. At the time of analysis, BrdUrd incorporated into DNA was labelled with primary BrdUrd antibodies followed by secondary FITC-conjugated antibodies, and DNA was stained with PI, as recently described (Silva et al. 2013). The labelled cells were analysed by flow cytometry using an Ortho Cytoron Absolute flow cytometer (Ortho Diagnostic Systems, Raritan, NJ, USA). The MultiCycle^®^ software program (Phoenix Flow Systems, CA, USA) was used for evaluation of the data with respect to DNA and BrdUrd contents. Cell cycle progression during the 24 h after seeding was monitored by following changes in relative movement zero (RM_zero_) and labelling index (LI) (Fredlund and Oredsson 1996).

### Western blot analysis of AzI

The cells were diluted in sample buffer (62.5 mM Tris–HCl, pH 6.8, 20 % glycerol, 2 % SDS, 5 % *β*-mercaptoethanol and 0.5 % bromophenol blue) and sonicated, followed by immediate boiling for 6 min. The samples were stored at −20 °C until analysis. Aliquots containing 50,000 or 100,000 cells were loaded and separated on 4–12 % acrylamide Bis–Tris gels (Invitrogen) using the Xcell Sure Lock™ Mini-Cell System (Invitrogen). The separated proteins were transferred to nitrocellulose membranes using the iBlot™ Dry Blotting System (Invitrogen). Thereafter, the membranes were blocked in 5 % non-fat dry milk in PBS-T (PBS containing 0.05 % Tween 20) and incubated overnight with the primary antibody against rat AzI (1:50,000) at 4 °C. Following washing in PBS-T, the membranes were incubated with the secondary horseradish peroxidase-conjugated goat anti-mouse IgG antibody (1:20,000) in PBS-T for 1 h at room temperature. The bands were detected with the ECL™ Advance Blotting Detection Kit. The ChemiDoc XRS system and the Quantity One software (both from Bio-Rad Laboratories Inc., Hercules, CA, USA) were used for imaging and data analysis. Actin was used as a loading control.

### Stable transfection of AzI

The coding region of human AzI1 was subcloned from pcDNA3.1-AzI (kindly provided by Leif Andersson, Helsinki) into the mammalian expression vector pCI-neo. Exponentially growing JIMT-1 and MCF-7 cells were transfected with the pCI-neo/AzI construct or the pCI-neo empty vector using a Gene Pulser^®^ II (Bio-Rad, CA, USA). Stable transfectants were selected by addition of geneticin (0.5 mg/ml) to the cell culture medium.

### Immunofluorescence microscopy

Cells were cultured on sterile poly-l-lysine-coated glass slides for 48 h. After fixation in 3.7 % paraformaldehyde (in PBS) for 15 min at room temperature and subsequent washing in PBS, the slides were blocked in 5 % non-fat dry milk in PBS-T for 1 h at room temperature. The cells were incubated with the primary AzI antibody diluted 1:10,000 in PBS-T overnight at 4 °C. After washing, the cells were incubated for 1 h with the Alexa Fluor 488 Goat Anti-mouse antibody (Invitrogen) (diluted 1:500 in PBS-T), at room temperature. Slides were counterstained with bisbenzimide and finally washed in PBS before mounting. Fluorescence-labelled cells were photographed using an Olympus/Nikon epifluorescence microscope (Olympus Optical Co. Ltd., Japan) equipped with a digital camera (Nikon Imaging Japan Inc., Japan). The final images shown were obtained by overlaying AzI- and bisbenzimide-stained pictures using Adobe Photoshop 8.0.1 (Adobe Systems Incorporated, San Jose, California, USA).

### Statistical analysis

Values are expressed as mean ± SEM. Students’ *t* test was used for statistical evaluation and *p* < 0.05 was considered as significant.

## Results

### Effects of DFMO on polyamine homeostasis

**Fig. 1:**
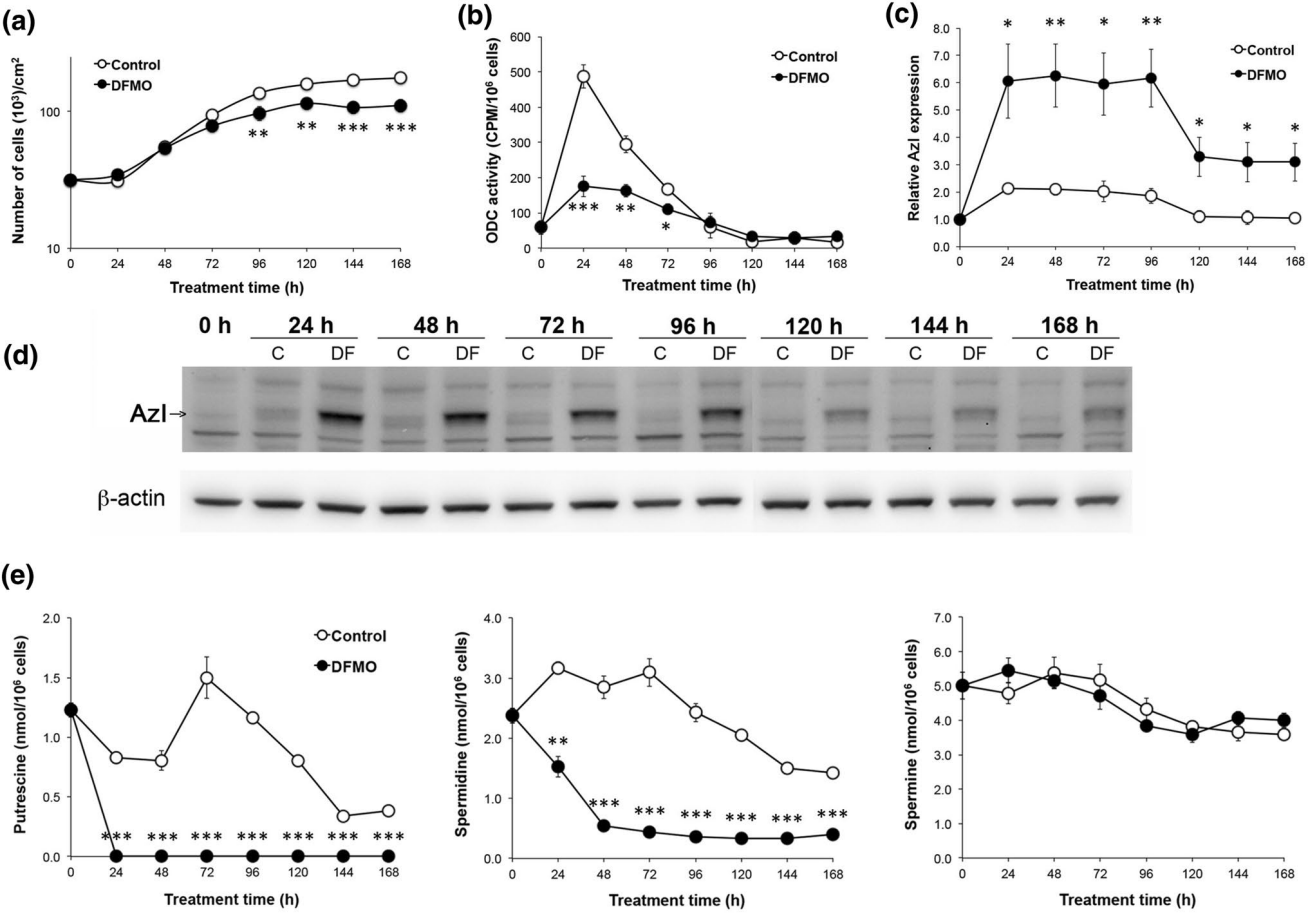
Long-term effects of DFMO on cell proliferation, ODC activity, AzI level and polyamine content in JIMT-1 cells. Cells were seeded in the absence or presence of 1 mM DFMO (DF). **a** The cells were harvested for analyses every 24 h for up to 168 h by trypsinisation and the cell number was determined by using a hemocytometer. Cell pellets were prepared and used for various analyses. **b** ODC activity was determined by a radiometric assay. **c** and **d** AzI was determined and quantified by Western blot (**d**) and the data from three experiments were densitometrically scanned and presented as relative AzI expression (**c**). **e** Putrescine, spermidine and spermine contents were determined by HPLC. Values are mean ± SEM (*n* = 3–7). When not visible, the *SEM bars* are covered by the *symbols*. *p < 0.05; **p < 0.01; ***p < 0.001 (compared to controls)

Initially, we analysed the effects of DFMO on JIMT-1 breast cancer cells. Treatment with DFMO significantly reduced the proliferation of JIMT-1 cells seeded in fresh medium (Fig. 1a). The late effect of DFMO on cell proliferation was preceded by changes in the polyamine metabolism, seen as early as 24 h after start of treatment (Fig. 1). ODC activity (which was transiently increased after reseeding) and intracellular polyamine content were decreased markedly by treatment with DFMO (Fig. 1b, e). In contrast to putrescine, which was depleted entirely after 24 h of DFMO treatment, the spermidine concentration continued to decrease to about 10 % of the control value at 48 h of treatment, whereas spermine was not affected significantly by DFMO treatment (Fig. 1e). Interestingly, the cellular content of AzI increased transiently after reseeding the cells in fresh medium (Fig. 1c, d). In parallel with the early changes in polyamine metabolism, the AzI level had increased dramatically after 24 h of DFMO treatment and remained elevated during the entire treatment cycle of 168 h, although a 50 % reduction was observed from day 5 (Fig. 1c, d).

### Effects of DFMO on cell cycle progression

**Fig. 2:**
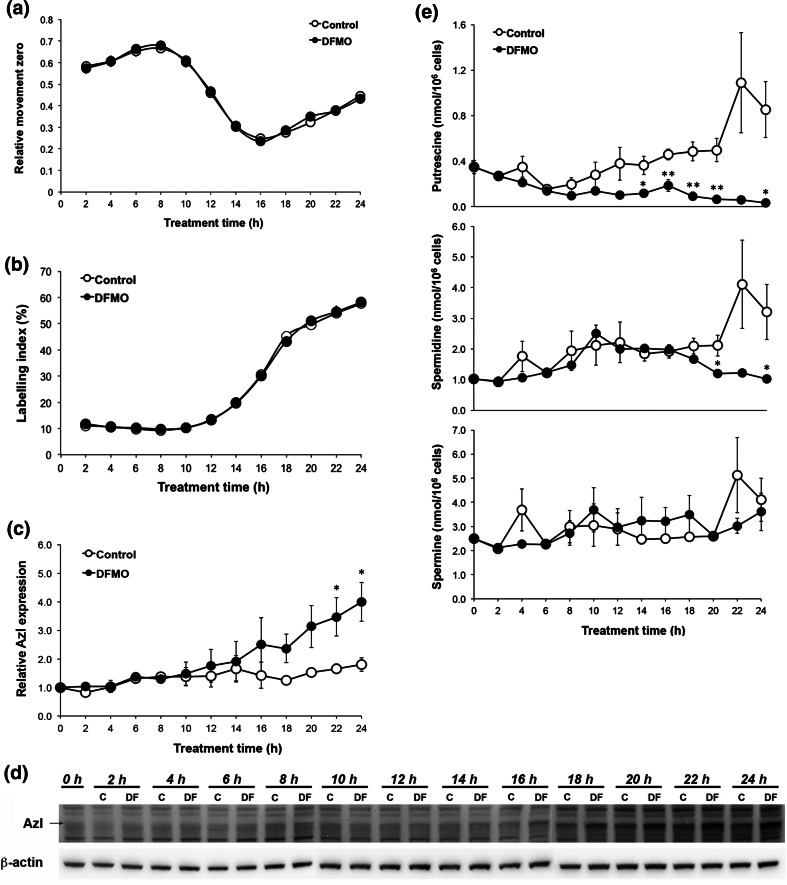
Short-term effects of DFMO on cell proliferation, AzI level and polyamine content in JIMT-1 cells. Cells were seeded in the absence or presence of 1 mM DFMO and sampled for various analyses every other hour for the first 24 h after seeding. A DNA—bromodeoxyuridine (BrdUrd) method was used to follow cell cycle progression after seeding. Thirty minutes prior to sampling, 5 µM BrdUrd was added to the medium of the cells. The cells were harvested and fixed in 70 % ethanol. After labelling DNA with PI and the incorporated BrdUrd with antibodies, the cells were analysed by flow cytometry. Cell cycle progression was monitored by following changes in RM_zero_ (**a**) and LI (**b**). AzI was determined by Western blot (**d**) and the data from three experiments were densitometrically scanned and presented as relative AzI expression (**c**). Putrescine, spermidine and spermine levels were determined by HPLC (**e**). Values are mean ± SEM (*n* = 4–5). When not visible, the *SEM bars* are covered by the *symbols*. *p < 0.05; **p < 0.01 (compared to controls)

The AzI content has been shown to vary during the cell cycle, with peaks in the G_1_ and G_2_/M phases (Murakami et al. 2009). Since the AzI content was increased significantly in JIMT-1 cells as early as 24 h of DFMO treatment, we decided to investigate whether this increase was a consequence of DFMO affecting the cell cycle progression. The cells used for seeding were partly synchronised and approximately 80 % (results not shown) were in G_0_/G_1_ phase (plateau phase cells). Small treatment-induced effects on the partially synchronised cell cycle progression may result in large changes in levels of proteins that vary during the cell cycle, like AzI. Thus, we determined whether DFMO provoked any changes in the cell cycle progression during the first 24 h of treatment. Cell cycle progression was followed by analyses of RM_zero_ and LI after labelling DNA with BrdUrd (Fig. 2a, b).

RM_zero_ is a measure of the distribution of BrdUrd-labelled cells in S phase (Begg et al. 1985; Fredlund and Oredsson 1996). When BrdUrd-labelled cells are distributed uniformly in S phase, RM_zero_ is 0.5. RM_zero_ is below 0.5 when the BrdUrd-labelled cells are in early S phase and above 0.5 when the cells are in late S phase. LI is a measure of the number of cells that have incorporated BrdUrd in proportion to the total number of cells examined. Figure 2a shows changes in RM_zero_ after seeding. RM_zero_ was above 0.5 at the first sampling time (i.e. 2 h after seeding) and then it increased to a maximum at 8 h after seeding. During this time, the LI was 10 % (Fig. 2b). Taken together, these data imply that the 10 % of cells in S phase were located mainly at the end of S phase at the time of seeding. The rapid drop in RM_zero_ between 8 and 16 h after seeding is caused by the massive inflow of cells from G_1_ phase into S phase, due to the partially synchronous proliferation of the cells (Fig. 2a). This is reflected in a large increase in LI during the same time period (Fig. 2b). DFMO treatment had no effect on this early cell cycle progression (Fig. 2a, b). Thus, the increased AzI level found after 24 h of treatment (Fig. 1c, d) was not caused by a DFMO-induced effect on early cell cycle progression. Instead, it is conceivable that the induction of AzI after treatment with DFMO was related to the decrease in cellular putrescine and/or spermidine content (Fig. 2e).

### Feedback control of AzI

**Fig. 3:**
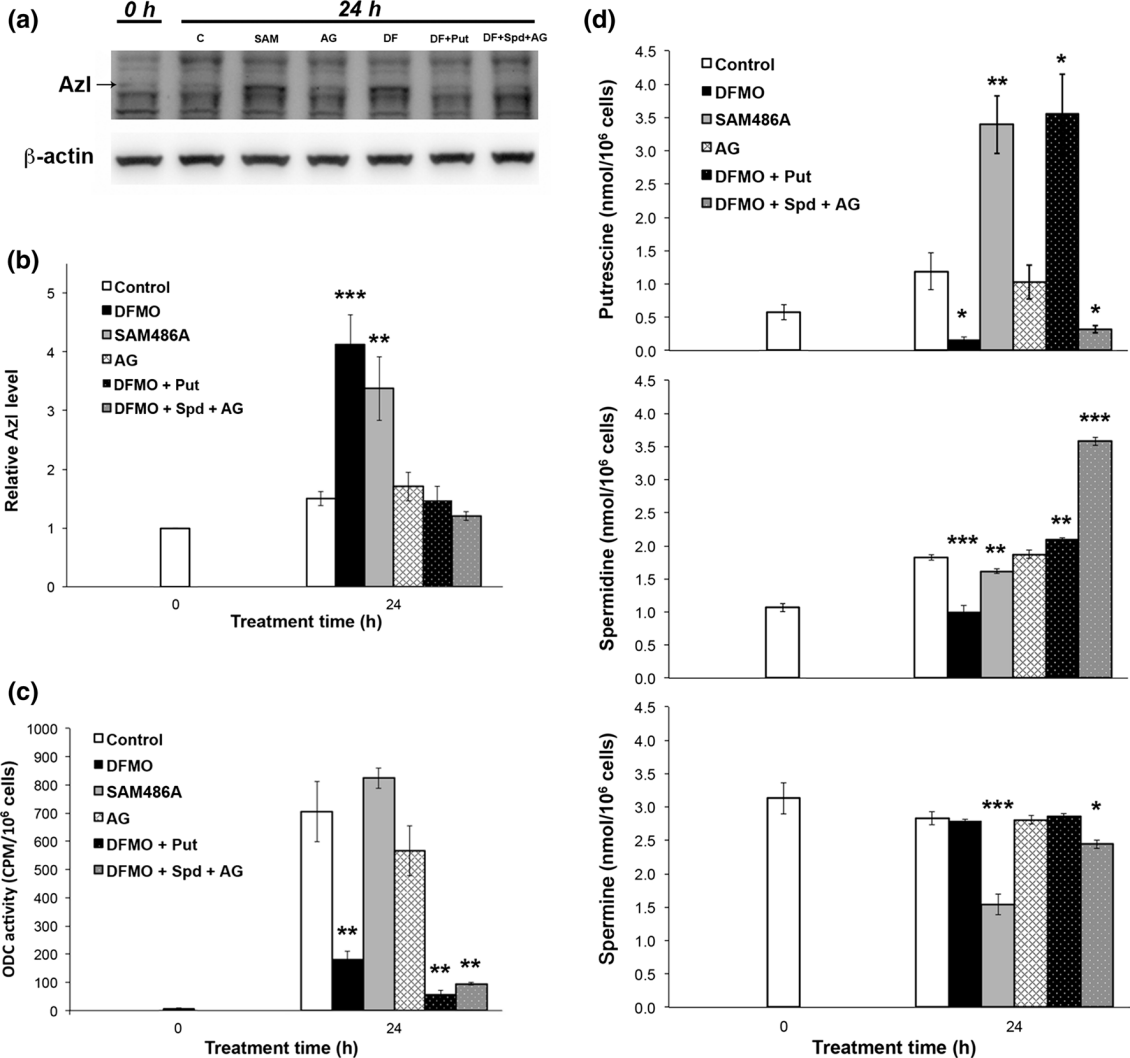
Regulation of AzI by polyamines in JIMT-1 cells. Cells were seeded in the absence of compound (control) or in the presence of 1 mM DFMO, 20 μM SAM486A, 1 mM aminoguanidine (AG), or 1 mM DFMO (DF) and 100 μM putrescine (put), or 1 mM DFMO, 50 μM spermidine (Spd) and 1 mM AG and then sampled at 24 h after seeding. AzI was determined by Western blot (**a**) and the data from three experiments were scanned using densitometry and presented as relative AzI expression (**b**). ODC activity was determined by a radiometric assay (**c**) and putrescine, spermidine and spermine contents (**d**) were determined by HPLC. Values are mean ± SEM (*n* = 3–6). When not visible, the *SEM bars* are covered by the *symbols*. *p < 0.05; **p < 0.01; ***p < 0.001 (compared to controls)

An extensive analysis of AzI expression during the first 24 h of DFMO treatment showed that the AzI level was significantly increased after 22 h of treatment (Fig. 2c, d), whereas the putrescine and spermidine levels were decreased at 14 and 20 h, respectively, after seeding in the presence of DFMO (Fig. 2e). To determine whether the induced increase in the cellular AzI level was due to the decrease in cellular putrescine and/or spermidine content caused by DFMO treatment, we examined if addition of putrescine or spermidine reversed the effect of the inhibitor on the AzI level seen after 24 h of treatment. As expected, addition of putrescine or spermidine to the cells treated with DFMO restored the cellular content of putrescine and/or spermidine to control or above control values (Fig. 3d). Moreover, the addition of putrescine or spermidine prevented the DFMO-induced increase in cellular AzI content, (Fig. 3a, b). AG, which was added to the growth medium to prevent extracellular oxidation of spermidine by serum amine oxidases, had no effect on AzI expression, ODC activity or polyamine content *per se* (Fig. 3). The effect of SAM486A on the cellular AzI level was also analysed. SAM486A is an inhibitor of S-adenosylmethionine decarboxylase, which together with ODC catalyses the key steps in the biosynthesis of polyamines (Pegg 2009). Treatment with SAM486A for 24 h resulted in an increased cellular level of AzI, which was similar to that observed after treatment with DFMO (Fig. 3a, b). The cellular putrescine content was also markedly increased, whereas the spermidine and particularly, the spermine content were decreased (Fig. 3d). Thus, the cellular expression of AzI appeared to be at least partly regulated by the polyamine pools. A decrease in the polyamine content thus resulted in an increase in AzI, which presumably caused an increase in the ODC level (due to the interaction of AzI with Az). Consequently, AzI is an important regulatory protein in the feedback control of polyamine homeostasis.

### Effects of AzI overexpression

**Fig. 4:**
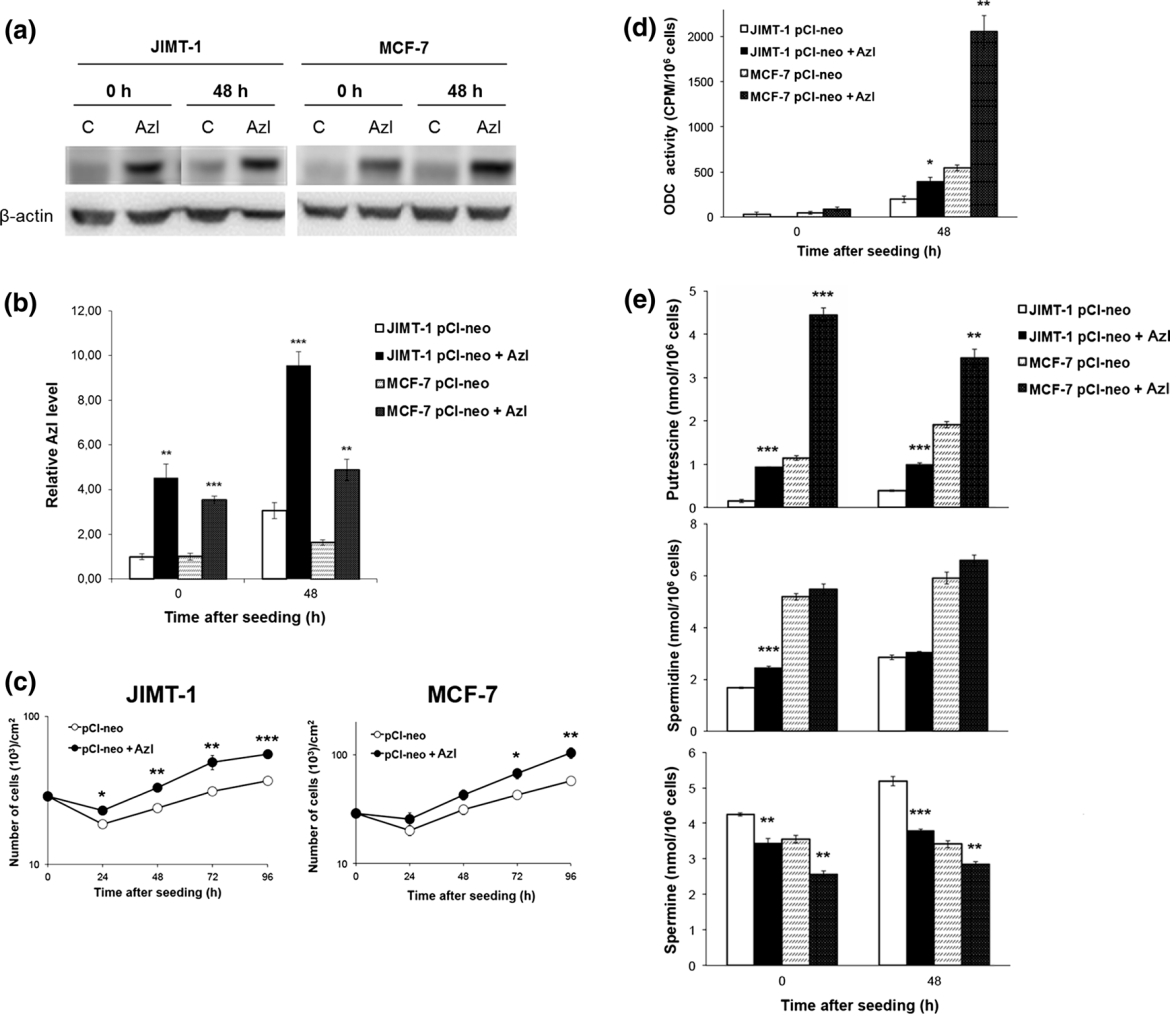
Effect of stable transfection with AzI in MCF-7 and JIMT-1 cells. MCF-7 and JIMT-1 breast cancer cells were stably transfected with empty vector (pCl-neo) or vector containing AzI (pCl-neo + AzI). AzI was determined by Western blot (**a**) and the data from three experiments were scanned using densitometry and presented as relative AzI expression (**b**). Proliferation of the cells was compared in growth curve experiments where the cell number was determined by counting in a hemocytometer (**c**). ODC activity was determined by a radiometric assay (**d**) and putrescine, spermidine and spermine contents (**e**) were determined by HPLC. Values are mean ± SEM (*n* = 3–6). When not visible, the *SEM bars* are covered by the *symbols*. *p < 0.05; **p < 0.01; ***p < 0.001 (compared to controls)

We next investigated the effect of perturbed AzI expression on polyamine homeostasis and cell proliferation. Two breast cancer cell lines, JIMT-1 and MCF-7, were transfected with an expression vector containing the coding region of human AzI1 and stable transfectants were isolated. As shown in Fig. 4a,b, both cell lines transfected with the AzI-containing vector exhibited a marked increase in the expression of AzI compared to the cells transfected with the empty vector. Interestingly, both cell lines expressing a high level of AzI also exhibited an increased cell proliferation over 96 h in culture, as compared to the control cells (Fig. 4c). Increased AzI expression was also associated with an increase in ODC activity in the transfected cells 48 h after seeding (Fig. 4d). This was seen clearly in the AzI-expressing MCF-7 cells, where a more than fourfold increase in ODC activity was observed compared to the control cells 48 h after seeding (Fig. 4d). The cellular content of putrescine increased markedly; whereas that of spermine decreased in the cells expressing high levels of AzI compared to the control cells (Fig. 4e). Expression of AzI did not affect cellular spermidine content (Fig. 4e).

### Cellular localisation of AzI

**Fig. 5:**
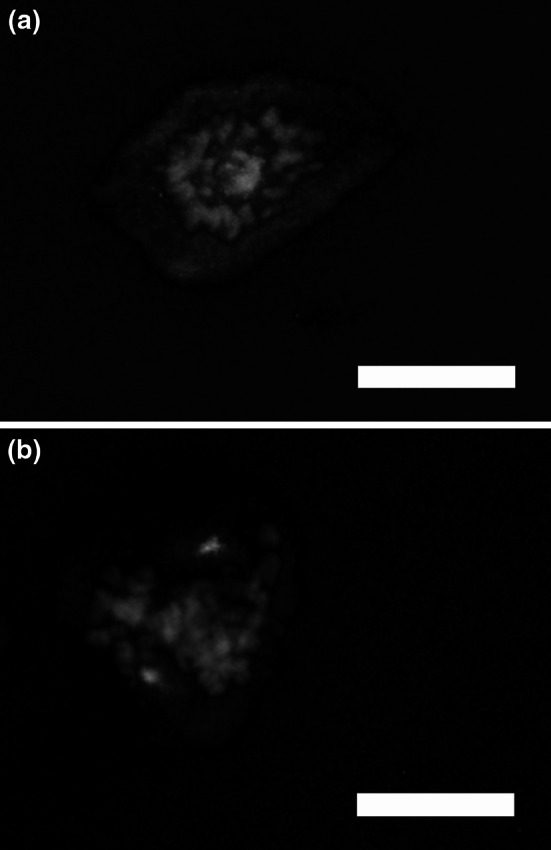
Localisation of AzI during mitosis in JIMT-1 cells. Cells were seeded on poly-l-lysine-coated glass slides and fixed in paraformaldehyde. They were then stained with the primary AzI antibody and the secondary Alexa Fluor 488 antibody (*green fluorescence*) and DNA was stained with bisbenzimide (*blue fluorescence*). (**a**) Early mitosis. (**b**) Metaphase/anaphase. Size of bar in fluorescence microscopy images: 20 µm

It has previously been reported that cellular localisation of AzI varies during the cell cycle, with a cytoplasmic localisation during interphase and a centrosomal localisation during mitosis, thereby indicating a role for AzI in the mitotic process (Mangold et al. 2008; Murakami et al. 2009). In the present study, we determined the intracellular localisation of AzI in JIMT-1 cells 48 h after seeding, using immunofluorescence microscopy (Fig. 5). In early mitosis, before chromosomal alignment and centrosomal separation, AzI was found in the cytoplasm and in the central part of the nuclear area (Fig. 5a). In metaphase/anaphase, AzI was localised in a pattern that resembled the two centrosomes having chromosomes in between (Fig. 5b).

## Discussion

As shown in the present study, the cellular level of AzI increased during the exponential growth of JIMT-1 cells. The increase probably reflects a rapid induction of AzI transcription after reseeding the cells in fresh medium. Nilsson et al. (2000) have demonstrated a rapid increase in AzI mRNA in mouse fibroblasts after growth stimulation by serum. Since binding of ODC to Az inhibits ODC activity, as well as stimulates its degradation, it is highly likely that the early increase in ODC activity seen in the JIMT-1 cells after seeding in fresh medium can be partly explained by a stabilisation/reactivation of the ODC protein caused by the sequestering of Az by AzI (Murakami et al. 1989; Nilsson et al. 2000). ODC activity started to decrease after 24 h, whereas the AzI level remained elevated for 96 h, after reseeding the cells in fresh medium. The ODC level (and activity) is dependent on a variety of factors which affect synthesis and degradation (e.g. ODC synthesis rate, levels of Az and AzI) and thus the ODC activity may decrease even though the level of AzI remains elevated.

We also demonstrate that DFMO inhibits the polyamine biosynthetic pathway, while simultaneously increasing the AzI level in exponentially growing breast cancer cells, indicating a feedback control of AzI by the polyamines. Since the AzI level has been reported to fluctuate within the cell cycle with peaks during early G_1_ and G_2_/M phases (Murakami et al. 2009), the observed increase in AzI level after DFMO treatment could also be due to an accumulation of cells in a specific phase (e.g. G_1_) of the cell cycle caused by the ODC inhibitor. However, it was clear that the cell cycle progression was not affected by DFMO during the first 24 h of treatment, whereas the induction of AzI was observed as early as 16 h after start of treatment. Instead, the increase in AzI caused by DFMO appeared to correlate with a decrease in putrescine and/or spermidine levels in the cells. This was further supported by the reversible effect of putrescine and spermidine on DFMO-induced increase of AzI. Moreover, treatment with SAM486A resulted in a similar increase in cellular AzI content as that observed after DFMO treatment. However, the effects of the two inhibitors on cellular polyamine content differed. DFMO reduced putrescine and spermidine levels, whereas SAM486A reduced spermidine and spermine levels along with an increase in putrescine content. Thus, it would appear that the negative control of AzI is not exerted by a single polyamine. Instead, all of the three polyamines may have the capability to down-regulate AzI, although with different potencies. Murakami et al. (2009) have previously shown that the expression of AzI is negatively regulated by any of the polyamines in HTC cells.

Ivanov et al. (2008) demonstrated that all available sequences of vertebrate AzI mRNAs contain an upstream conserved coding region (uCC) of about 50 codons. The uCC lacks an in-frame AUG codon, but contains a conserved AUU near the 5′ end, which might serve as an initiation codon for the uCC. Using a luciferase reporter assay, Ivanov et al. (2008) were able to show that the uCC of mouse AzI mRNA mediates polyamine-induced repression of the downstream main open reading frame. In spermidine-supplemented cells, the expression of the main open reading frame was repressed 6.5-fold compared with that of polyamine-depleted (DFMO-treated) cells. However, this repression was essentially lost when the putative initiation codon of the uCC was mutated from AUU to a non-initiating UUU codon. Moreover, mutating the last 10 sense codons of the uCC gave similar results, eliminating polyamine-induced repression of main open reading frame translation. The results obtained in the present study together with those of Murakami et al. (2009), confirm that the expression of AzI in mammalian cells is regulated by polyamines. It is highly likely that this regulation occurs, at least partly, at the translational level by the mechanism suggested by Ivanov et al. (2008). AzI is reported to have a short half-life (Bercovich and Kahana 2004) and thus any change in the synthesis rate will result rapidly in a change in the level of the AzI protein. Murakami et al. (2009) were able to show that putrescine down-regulated AzI mainly at the translation level in HTC cells. Murakami et al. (2014) recently demonstrated that polyamines may also regulate AzI expression by affecting the transcription as well as the splicing pattern of the mRNA. They showed that polyamines, besides reducing the transcription of full-length AzI1 mRNA, actually increased the level of a splice variant with a premature termination codon (coding for an AzI lacking almost two-thirds of the C-terminal region). In addition, polyamine depletion achieved after treatment with DFMO gave the opposite effect.

Increased levels of ODC and polyamines are distinctive features of rapid cell proliferation and of numerous forms of cancer (Wallace et al. 2003; Pegg 2006). ODC may be considered as a possible oncogene and overexpression of this enzyme has been demonstrated to induce cellular transformation in a variety of systems (Wallace et al. 2003; Pegg 2006; Shantz and Levin 2007). Thus, polyamine homeostasis is highly regulated and AzI appears to play an important role in this regulation. As shown in the present study, overexpression of AzI resulted in an increased cell proliferation, along with elevated levels of ODC and putrescine. Interestingly, the marked increase in ODC activity seen in the AzI-transfected cells during exponential growth (48 h after seeding) was not correlated with any major changes in polyamine levels, indicating that polyamine homeostasis is dependent on a variety of factors other than ODC activity [e.g. substrates for synthesis, other enzymes in the polyamine pathway (synthetic or degradative), cellular uptake or efflux of polyamines]. The increase in ODC activity was most likely related to a stabilisation of the enzyme due to the binding of Az to AzI. Keren-Paz et al. (2006) demonstrated that NIH3T3 mouse fibroblasts overexpressing AzI had elevated ODC and polyamine levels, and proliferated faster than control cells. Similar findings were obtained by Kim et al. (2006) using NIH3T3 cells and AT2.1 rat prostate carcinoma cells overproducing AzI. Keren-Paz et al. (2006) also demonstrated that AzI-overexpressing cells grew in the presence of low concentrations of serum, formed colonies in soft agar and gave rise to tumours when injected into nude mice, which all are attributes of transformed cells. In addition, AzI has been found to be up-regulated in a large number of cancers and thus may be regarded as a putative oncogene (Jung et al. 2000; Schaner et al. 2003; van Duin et al. 2005; Chin et al. 2007; Olsen and Zetter 2011). Conversely, silencing of AzI expression has been shown to reduce cell proliferation in vitro (Choi et al. 2005; Keren-Paz et al. 2006; Kim et al. 2006; Olsen et al. 2012), as well as repress tumour growth in vivo (Olsen et al. 2012). The effects of AzI most likely occur through the binding to Az and suppression of its functions. In addition to affecting the turnover of ODC, Az has been reported to stimulate the degradation of proteins important for cell cycling as well as centrosome duplication (Newman et al. 2004; Kasbek et al. 2010; Dulloo et al. 2010).

The results of the present study support the interesting observation of a centrosomal localisation of AzI during mitosis previously reported by Mangold et al. (2008) and Murakami et al. (2009). Also Az has been shown to be co-localised with the centrosome during mitosis (Mangold et al. 2008; Murakami et al. 2009). Interestingly, alterations in the AzI/Az ratio caused abnormalities in numbers of centrosomes in the cell, which further indicates a role for Az (and AzI) in centrosomal duplication (Mangold et al. 2008). It is conceivable that Az regulates the turnover of an essential centrosomal component. In fact, Az has been reported to stimulate the degradation of Aurora-A, which is a key protein in centrosome amplification (Lim and Gopalan 2007). However, more work is needed to identify the exact functions of Az and AzI in mitosis.

In conclusion, our findings strongly indicate that AzI plays essential roles in polyamine homeostasis and cell proliferation.

## Abbreviations

AG: Aminoguanidine
Az: Antizyme
AzI: Antizyme inhibitor
BrdUrd: Bromodeoxyuridine
DFMO: Difluoromethylornithine
ECL: Enhanced chemiluminescence
FITC: Fluorescein isothiocyanate
LI: Labelling index
ODC: Ornithine decarboxylase
PBS: Phosphate-buffered saline
PI: Propidium iodide
RM_zero_: Relative movement zero
uCC: Upstream conserved coding region
